# Attenuation of inhibitory PAS domain protein-induced cell death by synthetic peptides derived from Mcl-1 transmenbrane domain

**DOI:** 10.1038/s41420-021-00475-3

**Published:** 2021-05-04

**Authors:** Shuya Kasai, Ken-ichi Yasumoto, Kazuhiro Sogawa

**Affiliations:** 1 Department of Biomolecular Sciences, Graduate School of Life Sciences, Tohoku University, Aoba-ku Sendai, 980-8578 Japan; 2 Present Address: Department of Stress Response Science, Center for Advanced Medical Research, Hirosaki University Graduate School of Medicine, Hirosaki, Japan

**Keywords:** Cell death in the nervous system, Apoptosis

## Abstract

Expression of Inhibitory PAS domain protein (IPAS) induces apoptosis by inhibiting the anti-apoptotic activity of mitochondrial pro-survival proteins including Bcl-x_L_ and Mcl-1 through direct binding. Analysis to examine the IPAS-binding region in Bcl-x_L_ demonstrated that the C-terminal transmembrane (TM) domain is indispensable for the specific binding. A chimeric protein composed of the TM domain of Mcl-1 fused to the C-terminus of Citrine also exhibited a binding affinity to IPAS, and markedly attenuated apoptosis caused by the overexpression of Cerulean-IPAS in SH-SY5Y cells. HIV-1 TAT cell-penetrating peptide-conjugated synthetic peptides that cover whole or parts of the Mcl-1 TM domain showed anti-apoptotic activity in the CoCl_2_-induced cell death in PC12 cells. Administration of these highly effective anti-apoptotic peptides to mice treated with 1-methyl-4-phenyl-1,2,3,6-tetrahydropyridine (MPTP) that produces a reliable mouse model of Parkinson’s disease (PD) decreased neuronal cell loss in the substantia nigra pars compacta. Therefore, the peptides may be considered promising therapeutic agents for neurodegenerative disorders such as PD and stroke.

## Introduction

Inhibitory PAS domain protein (IPAS) has been revealed as a bifunctional protein. It not only suppresses the transactivation activity of hypoxia-inducible factor 1^1^ but is also involved in the mitochondrial pathway of apoptosis^2^. IPAS was transcriptionally upregulated by oxidative stress-induced and cytokine-induced NF-κB activation, leading to cell death^2,3^. We previously demonstrated that IPAS was involved in neurodegeneration in a 1-methyl-4-phenyl-1,2,3,6-tetrahydropyridine (MPTP)-induced mouse model of Parkinson’s disease (PD), and degraded by activation of the PINK1-Parkin pathway^4^. The pro-apoptotic activity of IPAS depends on direct binding to pro-survival proteins including Bcl-x_L_, Bcl-w, and Mcl-1 by which their binding activity to Bax was inactivated^2^. Phosphorylation of IPAS by stress-activated MK2 augmented its pro-apoptotic activity by enhancing the binding affinity to Bcl-x_L_^5^. These molecular mechanisms of apoptosis induction by IPAS are reminiscent of the mechanisms that Bcl-2 homology 3 (BH3)-only proteins cause apoptosis^6,7^. However, the BH3 motif, L-x-x-x-G-D-E (x = any amino acid), that is conserved in BH3-only proteins were not found in IPAS^2^. This motif forms an amphipathic alpha-helix to which a hydrophobic cleft formed by BH1, BH2, and BH3 domains of pro-survival proteins can bind, leading to initiation of apoptosis^8^. Thus, the absence of the motif in IPAS suggested that a different binding mechanism was involved in the association between IPAS and pro-survival proteins.

In this study, we demonstrate that IPAS directly binds to the transmembrane (TM) domain of Bcl-x_L_ and Mcl-1. Cell-penetrating HIV-1 TAT-conjugated synthetic peptides containing parts of the Mcl-1 TM sequence showed anti-apoptotic properties in CoCl_2_–induced apoptosis in PC12 cells. We also describe that these peptides attenuate cell loss of tyrosine hydroxylase (TH)-positive neurons in the substantia nigra pars compacta (SNpc) of mice treated with MPTP which is most widely used to produce animal models of PD.

## Results and discussion

### IPAS-binding region in Bcl-x_L_ and Mcl-1

**Fig. 1:**
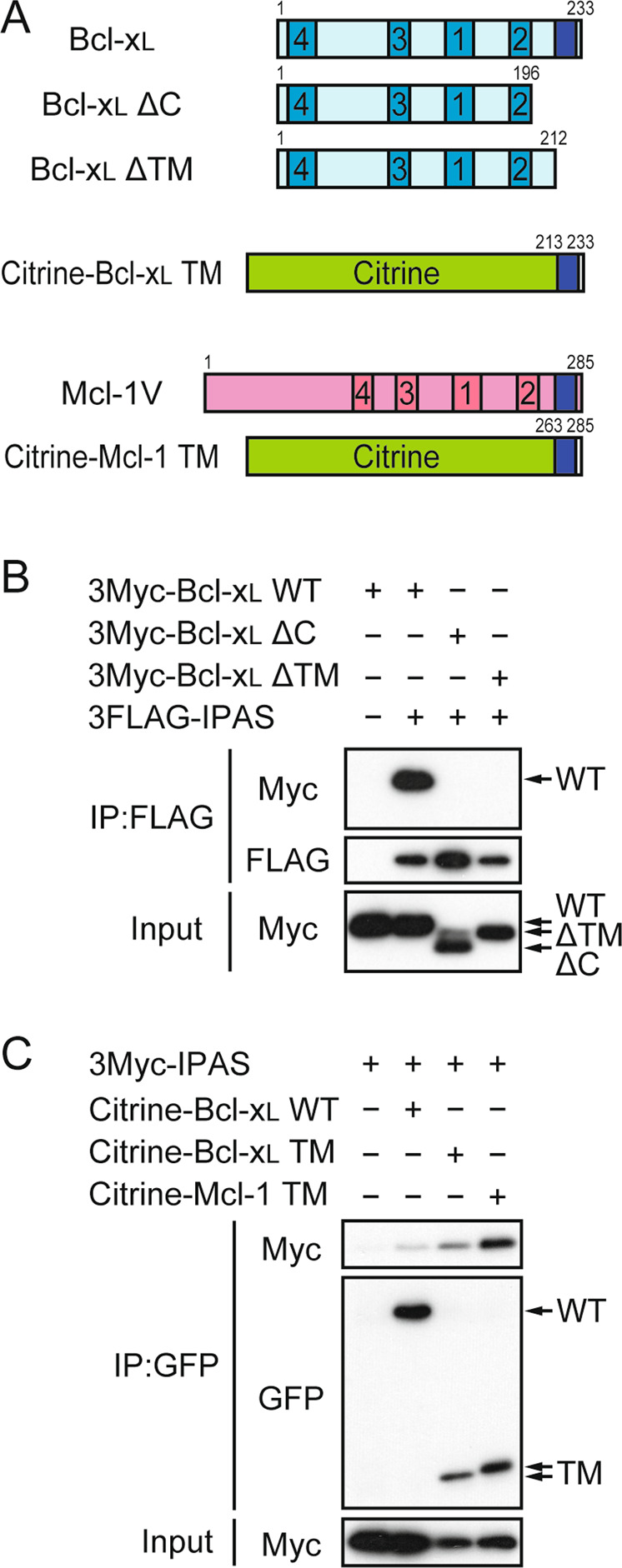
Binding of IPAS to the TM region of Bcl-x_L_ and Mcl-1. **A** Schematic representation of the structure of Bcl-x_L_, Mcl-1V, and their deletion mutants. Bcl-2 homology domains, BH1-4, and TM regions were indicated by numbered and dark blue boxes, respectively. **B** Lack of binding of tail-less Bcl-x_L_ to IPAS. HEK293T cells were transfected either with pBOS-3FLAG-IPAS and pBOS-3Myc-Bcl-x_L_ WT, pBOS-3FLAG-IPAS, and ΔC or pBOS-3FLAG-IPAS and ΔTM as described in “Materials and Methods” section. Twenty-four hours after transfection, cellular proteins were extracted and subjected to immunoprecipitation using the antibody against FLAG, and bound 3Myc-Bcl-x_L_ was analyzed by immunoblotting. **C** Binding of Bcl-x_L_ and Mcl-1 TM regions to IPAS. 3Myc-IPAS was coexpressed either with Citrine-Bcl-x_L_ TM or Citrine-Mcl-1 TM in HEK293T cells and analyzed as in **B**.

Bcl-x_L_ consists of four BH domains and a C-terminal TM anchoring domain (Fig. 1A). We expressed in HEK293T cells a tail-less mutant (Bcl-x_L_ ΔC) of Bcl-x_L_ lacking C-terminal 37 amino acids, which is dispensable for binding to BH3-only proteins, and examined its binding ability to IPAS. Surprisingly, the deletion mutant was unable to bind to IPAS (Fig. 1B). Furthermore, a mutant (Bcl-x_L_ ΔTM) with a shorter deletion of C-terminal 21 amino acids that only cover the TM domain also showed no detectable binding to IPAS. Next, we investigated the binding ability of the TM domain to IPAS by expressing a chimeric protein containing the TM domain fused to the C-terminus of Citrine (a yellow variant of GFP) (Fig. 1C). The protein exhibited binding activity towards IPAS. A similar construct containing the TM domain of Mcl-1 and two amino acids flanking the domain also showed marked binding to IPAS.

### Inhibition of IPAS-induced cell death by the Mcl-1 TM domain

**Fig. 2:**
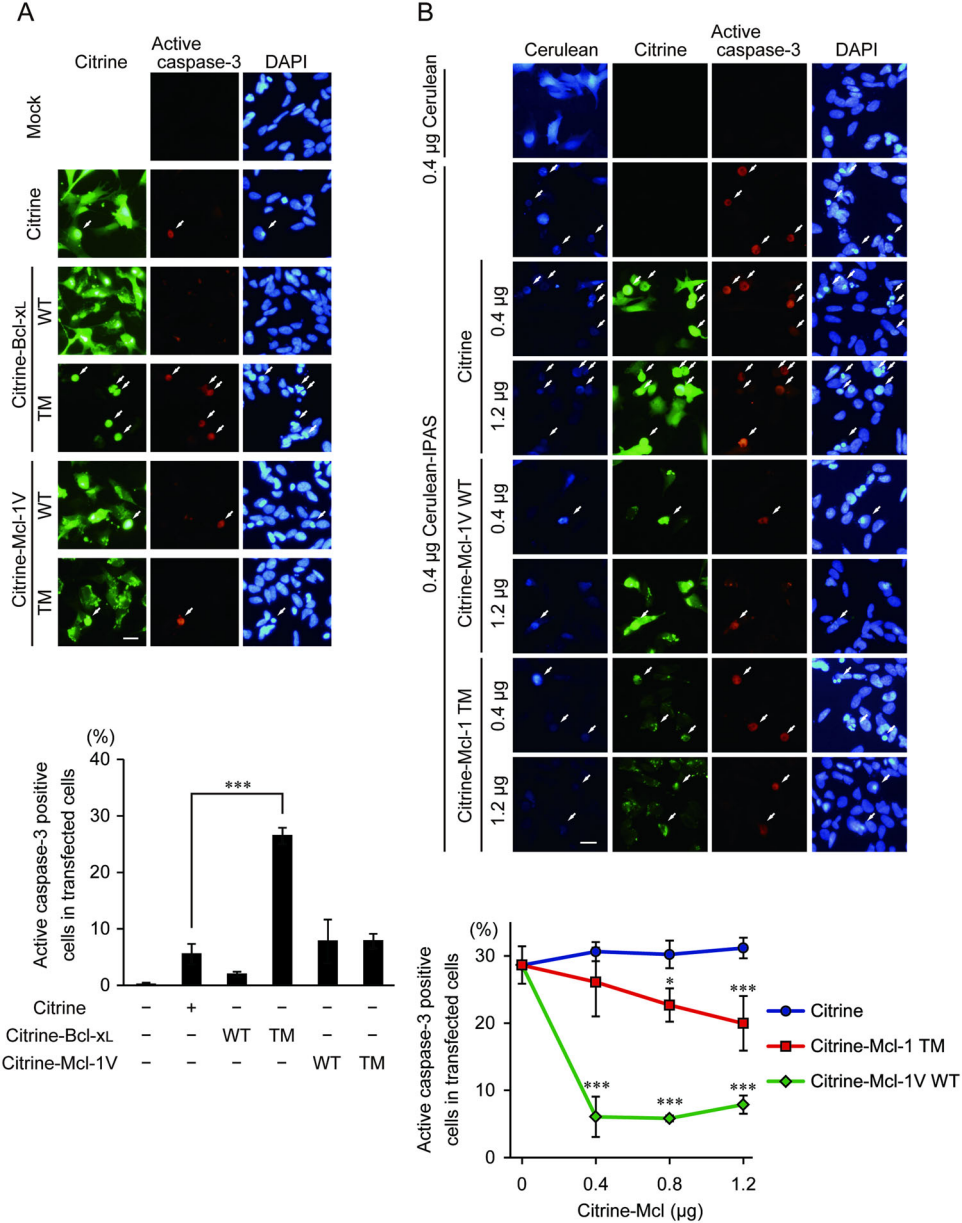
Suppression of IPAS-induced apoptosis by coexpression of Mcl-1 TM. **A** Cell toxicity of Bcl-x_L_ TM peptide. SH-SY5Y cells in a 12-well plate were transfected with 1.6 μg pCitrine-C1, pCitrine-Bcl-x_L_ WT, TM, pCitrine-Mcl-1 WT, or TM, and fixed 24 h after transfection. Apoptotic cells were stained by antibody against active caspase-3. Active caspase-3-positive cells were indicated by the arrows. Scale bar, 20 μm. The bar graph below represents the percentage of active caspase-3 positive cells detected by the observation of at least 300 transfected cells (mean ± SD of three independent experiments). ***p < 0.001. **B** Decrease in Cerulean-IPAS induced cell death by the expression of Citrine-Mcl-1V WT or Mcl-1TM. SH-SY5Y cells in a 12-well plate were cotransfected with 0.4 μg of pCerulean-C1 or pCerulean-IPAS and 0.4–1.2 μg of pCitrine-C1, pCitrine-Mcl-1V WT, or pCitrine-Mcl-1 TM. Apoptotic cells were analyzed as in **A**. Scale bar, 20 μm. The line graph below represents the percentage of active caspase-3 positive cells detected by the observation of at least 300 transfected cells (mean ± SD of four independent experiments). *p < 0.05, ***p < 0.001.

We transiently expressed Cerulean (a cyan variant of GFP)-IPAS in SH-SY5Y cells to induce apoptosis as described^4^, and investigated the cell-protection effect of the TM domains. Although expression of full-length Bcl-x_L_ fused to Citrine (Citrine-Bcl-x_L_ WT) without coexpression of Cerulean-IPAS showed no damaging effect on the cells as assessed by immunofluorescent staining of active caspase-3, a single expression of the TM domain of Bcl-x_L_ fused to Citrine (Citrine-Bcl-x_L_ TM) caused enhanced cell death (Fig. 2A). On the other hand, expression of the Citrine-Mcl-1TM exhibited little effect on cell survival. We, therefore, investigated the cell-protection activity of Citrine-Mcl-1 TM against apoptotic cell death caused by Cerulean-IPAS. As shown in Fig. 2B, IPAS-induced cell death was dose-dependently decreased by the coexpression of Citrine-Mcl-1 TM. However, its protective effect was considerably low when compared with that of full-length Mcl-1V (an Mcl-1 isoform that has the same TM sequence as that of Mcl-1). The cause of the low activity was not known, but it suggests that some other parts in the N-terminal cytoplasmic region of Mcl-1V may play a protective role in IPAS-induced cell death, possibly by assisting the binding process between the TM region and IPAS.

### Inhibition of CoCl_2_-induced cell death in PC12 cells by TAT-Mcl-TM peptides

**Fig. 3:**
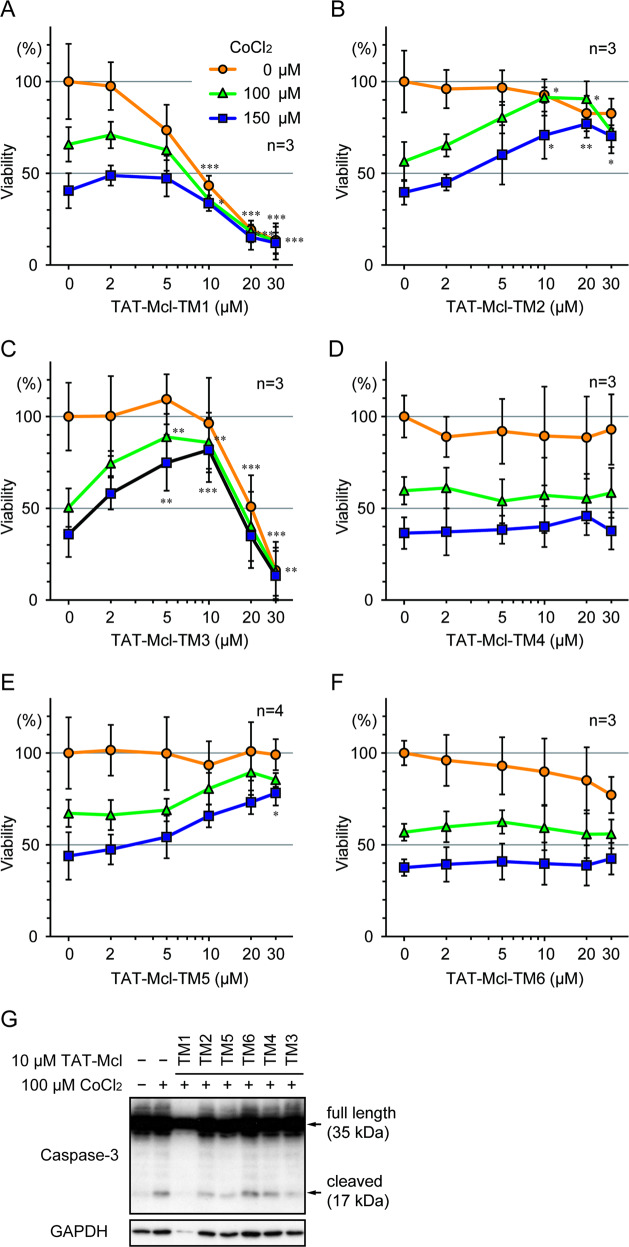
Inhibition of CoCl_2_-induced apoptosis in PC12 cells by the treatment with TAT-Mcl-TM peptides. **A**–**F** Viability of CoCl_2_-treated PC12 cells pretreated with TAT-Mcl-TM-derived peptides. PC12 cells were transduced with 2–30 μM TAT-Mcl-TM1 (**A**), TM2 (**B**), TM3 (**C**), TM4 (**D**), TM5 (**E**), or TM6 (**F**) for 2 h. Media were replaced with fresh media containing 0, 100, or 150 μM CoCl_2_, and cells were incubated for 16 h. Cell viability was determined by MTT assay, and data are expressed as mean ± SD from at least three independent experiments. *p < 0.05, **p < 0.01, ***p < 0.001. **G** Reduction of caspase-3 activation in CoCl_2_-treated PC12 cells by TAT-Mcl-TM peptides. PC12 cells were transduced with TAT-Mcl-TM peptides (10 μM) for 2 h and then treated with 100 μM CoCl_2_ for 16 h. Cellular proteins were extracted from whole cells and subjected to immunoblotting to detect intact (35 kDa) and cleaved active caspase-3 (17 kDa). GAPDH was detected as the loading control.

We previously reported that apoptosis in PC12 cells caused by CoCl_2_ treatment was IPAS-dependent^2^. Using the cell-based system, we evaluated with the MTT assay the anti-apoptotic activity of peptides derived from the Mcl-1 TM domain which were conjugated at its N-terminus to the HIV-1 TAT-derived cell-penetrating peptide. The TAT-Mcl-TM1 peptide comprising amino acids −2 to 21 (Table 1, the N-terminal asparagine residue of the TM domain was numbered as 1) showed a modest protecting activity at concentrations around 2 µM, although it was not statistically significant (Fig. 3A). At higher concentrations, it exhibited toxic effects on the cells. The cytotoxicity of the peptide against HeLa cells was similarly found but very weak against HEK293T cells, suggesting that the cytotoxicity was cell-type specific (Supplementary Fig. 1). TAT-Mcl-TM2 containing only the TM domain showed significant cell protecting activity at higher peptide concentrations (10–30 µM) (Fig. 3B). TAT-Mcl-TM5 with deletion of C-terminal 6 amino acids from TAT-Mcl-TM2 also showed a similar protection activity (Fig. 3E). Other deletion peptides (TAT-Mcl-TM4 and TAT-Mcl-TM6) of TAT-Mcl-TM2 exhibited no protective effects on the CoCl_2_-treated cells (Fig. 3D, F). TAT-Mcl-TM3 containing amino acids −2 to 15 showed a tendency of cell protection at around 2 µM and significantly protected cells at concentrations in the range of 5–10 µM while it caused cell death at higher concentrations (Fig. 3C).

**Table 1:** Amino acid sequence of TAT-Mcl-TM peptides.

Peptide sequences underlined are derived from the Mcl-1 transmembrane region.

Inhibitory effects of these TAT-Mcl-TM peptides on the activation of caspase-3 in CoCl_2_-treated PC12 cells were investigated (Fig. 3G). Caspase-3 activation was weakly found even in untreated cells, and it was increased by the CoCl_2_-treatment. The activation was inhibited by the addition of TAT-Mcl-TM2, 3 and 5 while inhibition by TAT-Mcl-TM4 and 6 was very weak. These results were in accordance with those obtained from the MTT assay. The activation of caspase-3 was not detected in the cells treated with TAT-Mcl-TM1 because protein recovery from the cells was extremely low presumably due to increased cell death. Cellular uptake of the peptides was confirmed by using FITC-labeled TAT-Mcl-TM3. Fluorescence from treated cells was detected in both the cytoplasm and nucleus (Supplementary Fig. 2).

Taken together, these results suggested that the cell protection activity against apoptosis caused by IPAS was localized to the first 15 residues of the TM domain and that the Ile-Arg sequence localized from −2 to −1 also played an important role. The addition of the two amino acids also created cytotoxicity against the PC12 cells. We previously reported that phosphorylation of IPAS at Ser184 enhanced binding affinity to Bcl-x_L_^5^. This phosphorylated Ser might interact with the Arg residue localized at position −1 because no other positively charged residues were present in the sequence found in this experiment.

### Attenuation of neuronal cell loss in the mouse model of PD by TAT-Mcl-TM peptides

**Fig. 4:**
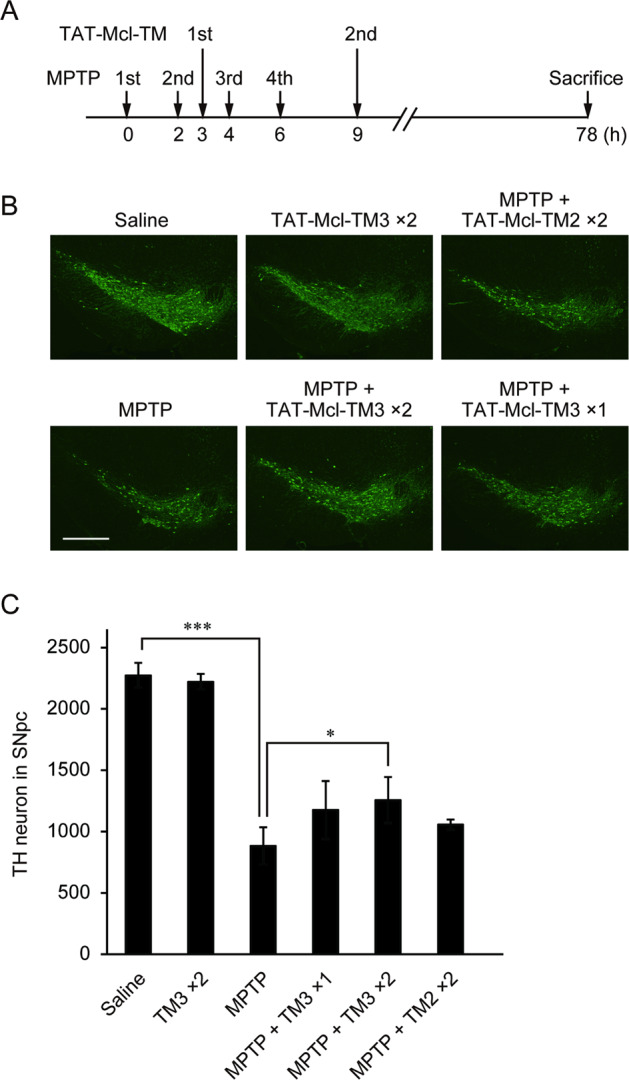
Protective effect of TAT-Mcl-TM peptides on MPTP-induced neurodegeneration. **A** Schedule of injections of MPTP and TAT-Mcl-TM peptides. C57BL/6 J mice were intraperitoneally injected 4 times with MPTP and 1 or 2 times of a TAT-Mcl-TM peptide (200 μg) at time points indicated by the arrows. **B, C** Decreased cell loss of TH-positive neurons in the SNpc of MPTP-injected mice by TAT-Mcl-TM peptides. Mice have administrated saline alone as a control (Saline), MPTP alone (MPTP), two doses of TAT-Mcl-TM3 (TM3×2), MPTP and a dose of TAT-Mcl-TM3 (MPTP + TM3×1), MPTP and two doses of TAT-Mcl-TM3 (MPTP + TM3×2), or MPTP and two doses of TAT-Mcl-TM2 (MPTP + TM2×2). Immunofluorescence analysis was performed using coronal sections through midbrains of treated mice. Every third section of each brain was immunostained for TH and observed with a fluorescence microscope. Representative images were shown (**B**). scale bar, 500 μm. The number of TH-positive neurons in the SNpc was scored and expressed as mean ± SD (**C**). *p < 0.05, ***p < 0.001.

We previously clarified that cell death of dopaminergic neurons in an MPTP mouse model of PD was partly IPAS-dependent^3^. Using the same model, the cell-protection activity of TAT-Mcl-TM peptides was investigated. The treatment schedule of MPTP-intoxicated male mice with TAT-Mcl-TM peptides is shown in Fig. 4A. A single intraperitoneal administration of TAT-Mcl-TM3 showed the tendency of protection toward MPTP-induced cell loss of tyrosine hydroxylase (TH)-positive neurons in the SNpc (Fig. 4B, C). Twice-daily intraperitoneal administration of the peptide at the 6 h time interval significantly attenuated the neuronal cell loss. A similar positive result was obtained when protective effects of TAT-Mcl-TM3 were examined in the different raising environments with elevated ambient temperature (Supplementary Fig. 3). We next examined the effect of TAT-Mcl-TM2 (Fig. 4B and C) on cell loss. It also exhibited a tendency of protection although it was not significant. Taken together, these results demonstrate that TAT-Mcl-TM peptides are protective agents against neurodegeneration in the SNpc caused by MPTP.

It was clarified that the C-terminal TM domain of Bcl-x_L_ and Mcl-1 was important for binding to IPAS (Fig. 1). This binding mode is totally different from that between BH3 only proteins and pro-survival Bcl-2 family proteins including Bcl-x_L_ and Mcl-1. In a survey of Bcl-x_L_-binding proteins, Praf2, a small transmembrane protein that may be involved in transport from the endoplasmic reticulum to the Golgi apparatus, was found^9^. Bcl-x_L_ also binds to Praf2 mainly through the TM domain of Bcl-x_L_, and through this specific interaction, Praf2 causes cerulenin-induced apoptosis in neuroblastoma cells. Similarly, the SARS-CoV protein 7a can induce caspase-dependent apoptosis in several cell lines via its interaction with the TM domain of Bcl-x_L_^10^. Apparent sequence similarity was not observed among IPAS, Praf2, and SARS-CoV protein 7a although they interact with the same TM sequence of Bcl-x_L_. There may be a large group of pro-apoptotic proteins that interact with the TM domain of pro-survival proteins and act in the early stages of the various apoptosis pathways. More extensive and detailed investigations are necessary to gain a further understanding of this type of pro-apoptotic protein.

IPAS is a downstream effector of NF-κB in neuronal apoptosis. Activation of NF-κB was found in the dopaminergic neurons of post-mortem PD brains^11^, and several studies reported that inhibition of the classical NF-κB activation pathway slowed the progression of PD in mouse and primate models^12^. In addition to PD, ample evidence was also found for an association between activation of NF-κB and progression of cerebral ischemia^13^. It has been reported that administration of TAT-conjugated Bcl-x_L_ protein has a protective effect against neuronal cell death in the mouse model of middle cerebral artery occlusion^14,15^. Although the studies did not identify a target molecule(s) of TAT-Bcl-x_L_ in the neuron, IPAS could be a potent target. Activation of NF-κB is also suggested in many neurodegenerative diseases such as Alzheimer’s disease (AD) and multiple sclerosis (MS)^16,17^. Mcl-TM peptides conjugated to cell-penetrating peptides may be useful for the treatment of these neurodegenerative diseases.

It would be possible to produce improved Mcl-TM peptides with higher affinity to IPAS and without cytotoxicity by introducing point mutations using natural and unnatural amino acids in the TM sequence. The binding of the TM domain to IPAS strongly suggests that a hydrophobic groove that accommodates hydrophobic TM helices is present on the surface of IPAS. Small-molecule inhibitors that fit the hydrophobic groove could also be used for the treatment of NF-κB-dependent neurodegenerative diseases.

## Materials and methods

### Reagents and antibodies

TAT-Mcl-TM1 peptide (the sequence is shown in Table 1) was synthesized as TFA salt by Peptide Institute (Osaka, Japan). The other peptides were synthesized as HCl salt by GenScript (Piscataway, NJ, USA). TAT-Mcl-TM peptides were dissolved in Milli Q water as concentrated as possible, handled in protein LoBind tubes (Eppendorf, Hamburg, Germany), and stored at −80 °C. MPTP and antibodies against FLAG and TH were obtained from Sigma-Aldrich (St. Louis, MO, USA). All other antibodies used were purchased from the following sources: anti-Myc (MBL, Nagoya, Japan); anti-Bcl-x_L_ (Cell Signaling Technology, Danvers, MA, USA); anti-caspase-3 (Promega, Fitchburg, WI, USA); anti-GFP (Clontech, Palo Alto, CA, USA).

### Plasmid construction

Plasmids for 3Myc-IPAS, 3FLAG-IPAS, Cerulean-IPAS, 3Myc-Bcl-x_L_, Citrine-Bcl-x_L_, and Citrine-Mcl-1V were constructed as described^2^. pBOS-3Myc-Bcl-x_L_ ΔC (1-196) and pBOS-3Myc-Bcl-x_L_ ΔTM (1-212) were constructed by PCR using mouse Bcl-x_L_ cDNA and following primers: Bcl-x_L_-1-F, 5’-AATTG ATATC ATGTC TCAGA GCAAC CGGG-3’; Bcl-x_L_-196R, 5’-AATTG ATATC TCACC CGTAG AGATC CACAA AAGT-3’, and Bcl-x_L_-212R, 5’-ATGCG ATATC TCAGC GGTTG AAGCG CTCCT-3’. pCitrine-Bcl-x_L_ TM (213-233) and pCitrine-Mcl-1 TM (263-285) were constructed by insertion of the following linkers into the Bgl II-EcoR I site of the pCitrine-C1 vector: Bcl-x_L_-213-233F, 5’-GATCT TGGTT CCTGA CGGGC ATGAC TGTGG CTGGT GTGGT TCTGC TGGGC TCACT CTTCA GTCGG AAGTG AG-3’; Bcl-x_L_-213-233R, 5’-AATTC TCACT TCCGA CTGAA GAGTG AGCCC AGCAG AACCA CACCA GCCAC AGTCA TGCCC GTCAG GAACC AA-3’; Mcl-1-263-285F, 5’-GATCT ATCAG GAATG TGCTG CTGGC TTTTG CAGGT GTTGC TGGAG TAGGA GCTGG TTTGG CATAT CTAAT AAGAT AGG-3’ and Mcl-1-263-285R, 5’-AATTC CTATC TTATT AGATA TGCCA AACCA GCTCC TACTC CAGCA ACACC TGCAA AAGCC AGCAG CACAT TCCTG ATA-3’. Constructions were confirmed by sequencing.

### Cell culture and DNA transfection

HEK293T, SH-SY5Y, PC12, and HeLa cells were obtained from the Cell Resource Center for Biomedical Research, Tohoku University, Sendai, Japan, and maintained as described previously^2,4^. HEK293T cells plated in 60-mm dishes were transfected with a mixture of two plasmids (1 μg each) using Lipofectamine 2000 (Invitrogen, Carlsbad, CA, USA) according to the manufacturer’s instructions.

### Immunoprecipitation and immunoblotting

At 24 h posttransfection, cells were harvested and cellular proteins were extracted and subjected to immunoprecipitation using an antibody against FLAG or GFP. The immunoprecipitates were analyzed by western blotting using the indicated antibodies as described previously^5^.

### MTT assay

HeLa cells were seeded at 10^4^ cells/well in a 96-well plate. PC12 and HEK293T cells were seeded at 4 × 10^4^ or 10^4^ cells/well, respectively, in PEI-coated 96-well plates. After overnight incubation, cells were treated with 2–30 μM peptides diluted in Opti-MEM for 2 h. Media were replaced with serum-containing RPMI 1640, and cells were treated with 100 or 150 μM CoCl_2_ for 16 h. After washing with PBS, cells were incubated in 0.5 mg/ml MTT (Dojindo, Kumamoto, Japan) in culture media for 3 h. MTT formazan was dissolved in dimethyl sulfoxide and photometrically quantified at 535 nm. The toxicity of peptides was calculated as described^18^.

### Animals

Male 9–12 weeks old C57BL/6J mice obtained from Japan SLC (Hamamatsu, Japan) were bred in a 12-h light/12-h dark cycle at 23 °C. All animal experiments were approved by the Committee for Animal Research of Tohoku University and performed in accordance with the Regulation for Animal Experiments and Related Activities as Tohoku University (Regulation No 122). Mice were injected 4 times with 15 mg/kg MPTP intraperitoneally at a 2-h interval. TAT-Mcl-TM peptides were diluted with saline and injected intraperitoneally at 3 h and 9 h after the first MPTP injection. Mice were sacrificed by inhalation of isoflurane 72 h after the last MPTP injection.

### Immunofluorescent staining

SH-SY5Y cells grown on coverslips in a 12-well plate were mock-transfected or transfected with 1.6 µg pCitrine-C1, pCitrine-Bcl-x_L_ WT, TM, pCitrine-Mcl-1V WT, or TM. Cells were fixed 24 h after transfection and stained with antibody against active caspase-3 as described previously^4^. Preparation of formalin-fixed paraffin-embedded brain sections and succeeding immunofluorescence staining of the sections using anti-TH antibody were carried out as described^4^.

### Statistical analysis

Multiple comparisons were analyzed by two-way ANOVA followed by post hoc Tukey-Kramer test.

## Supplementary information

Attenuation of Inhibitory PAS domain protein-induced cell death by synthetic peptides derived from Mcl-1 transmenbrane domain

Supplementary Figure 1

Supplementary Figure 2

Supplementary Figure 3
