# Supplementary material for: Attenuation of inhibitory PAS domain protein-induced cell death by synthetic peptides derived from Mcl-1 transmenbrane domain
Source: Cell Death Discov. 2021 May 4;7:92. doi: 10.1038/s41420-021-00475-3 (PMC8093901; doi:10.1038/s41420-021-00475-3)
Supplement: Supplementary file 1 — Attenuation of Inhibitory PAS domain protein-induced cell death by synthetic peptides derived from Mcl-1 transmenbrane domain [file 41420_2021_475_MOESM1_ESM.doc]

**Supplementary information**

**Attenuation of Inhibitory PAS domain protein-induced cell death by synthetic peptides derived from Mcl-1 transmenbrane domain**

Shuya Kasai, Ken-ichi Yasumoto & Kazuhiro Sogawa*

**Figure legends**

**Supplementary Fig. 1** **Cytotoxicity of TAT-Mcl-TM peptides.** PC12 (**A**), HeLa (**B**) or HEK293T (**C**) cells were treated with 2-30 μM TAT-Mcl-TM1, TM2 or TM3 for 2 h. Cell viability was analyzed 16 h later as described in Fig. 3. Concentration of peptide which caused 50% toxicity was indicated with dashed line.

**Supplementary Fig. 2 Cellular distribution of FITC-TAT-Mcl-TM3.** PC12 cells were treated with 10 µM FITC-TAT-Mcl-TM3 for 2 h and then incubated with medium containing 0.2 µg/ml Hoechst for 1 h. Scale bar, 20 µm.

**Supplementary Fig. 3** **Protective effect of TAT-Mcl-TM3 on MPTP-induced neurodegeneration in SNpc of mice raised in warmed bed chips.** **A** Schedule of injections of MPTP and TAT-Mcl-TM peptides. C57BL/6J mice were intraperitoneally injected 4 times with MPTP and 2 times of TAT-Mcl-TM3 at time points indicated by the arrows. The thick red line indicates the time　period during which bedding chips were kept at 27˚C by a heated mattress pad. **B, C** Decreased cell loss of TH-positive neurons in the SNpc of MPTP-injected mice by TAT-Mcl-TM3. Mice were administrated saline alone as a control (Saline), MPTP alone (MPTP), or MPTP and two doses of TAT-Mcl-TM3 (MPTP + TM3×2). Immunofluorescence analysis was performed using coronal sections through midbrains of treated mice. Every third section of each brain was immunostained for TH and observed with a fluorescence microscope. Representative images were shown (**B**). Scale bar, 500 μm. Number of TH-positive neurons in the SNpc was scored, and expressed as mean ± SD (**C**). **p* < 0.05, ****p* < 0.001.
