# Supplementary figures and images for: Attenuation of inhibitory PAS domain protein-induced cell death by synthetic peptides derived from Mcl-1 transmenbrane domain
Source: Cell Death Discov. 2021 May 4;7:92. doi: 10.1038/s41420-021-00475-3 (PMC8093901; doi:10.1038/s41420-021-00475-3)

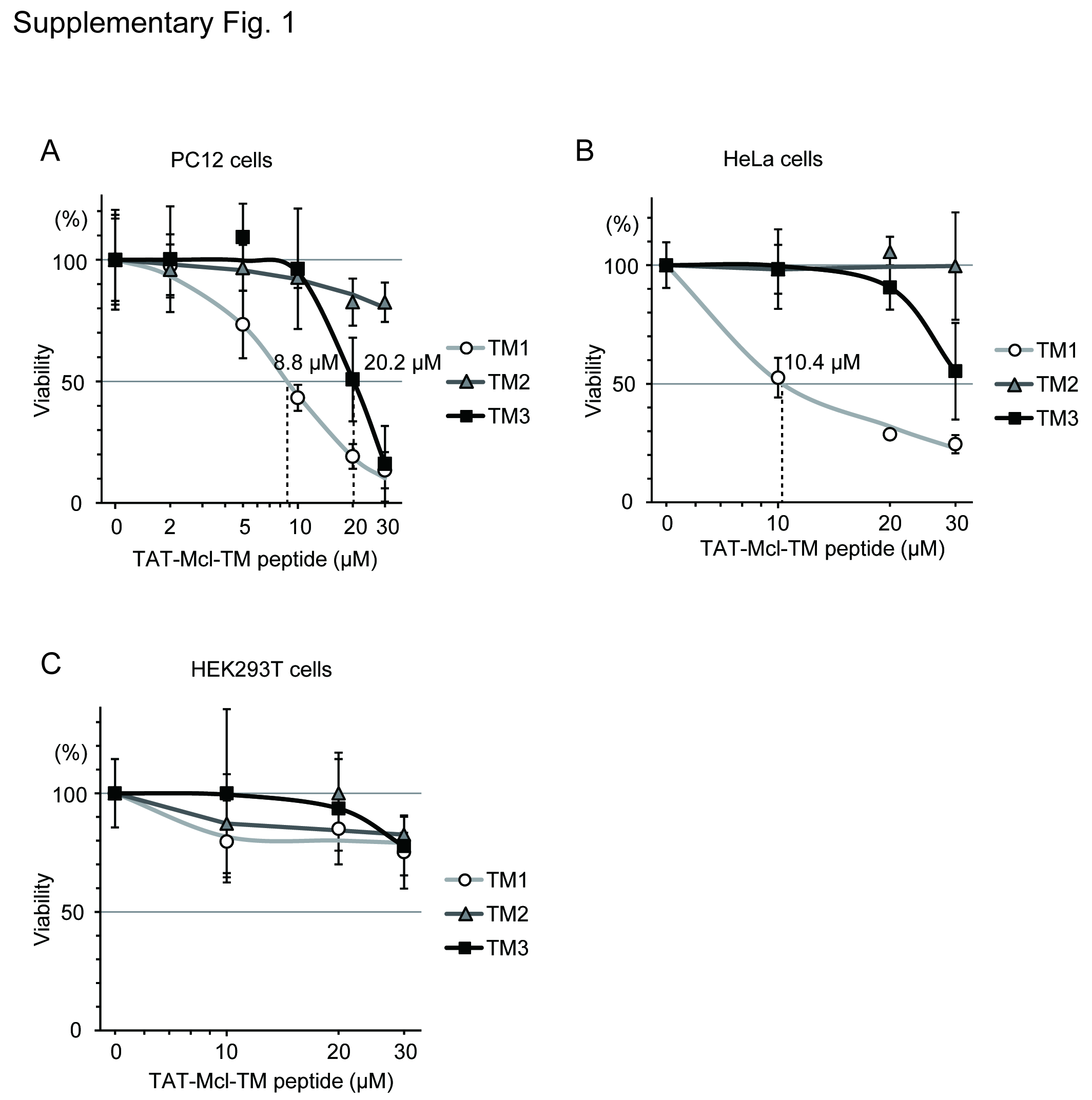

Supplement: Supplementary file 2 — Supplementary Figure 1 [file 41420_2021_475_MOESM2_ESM.tif]

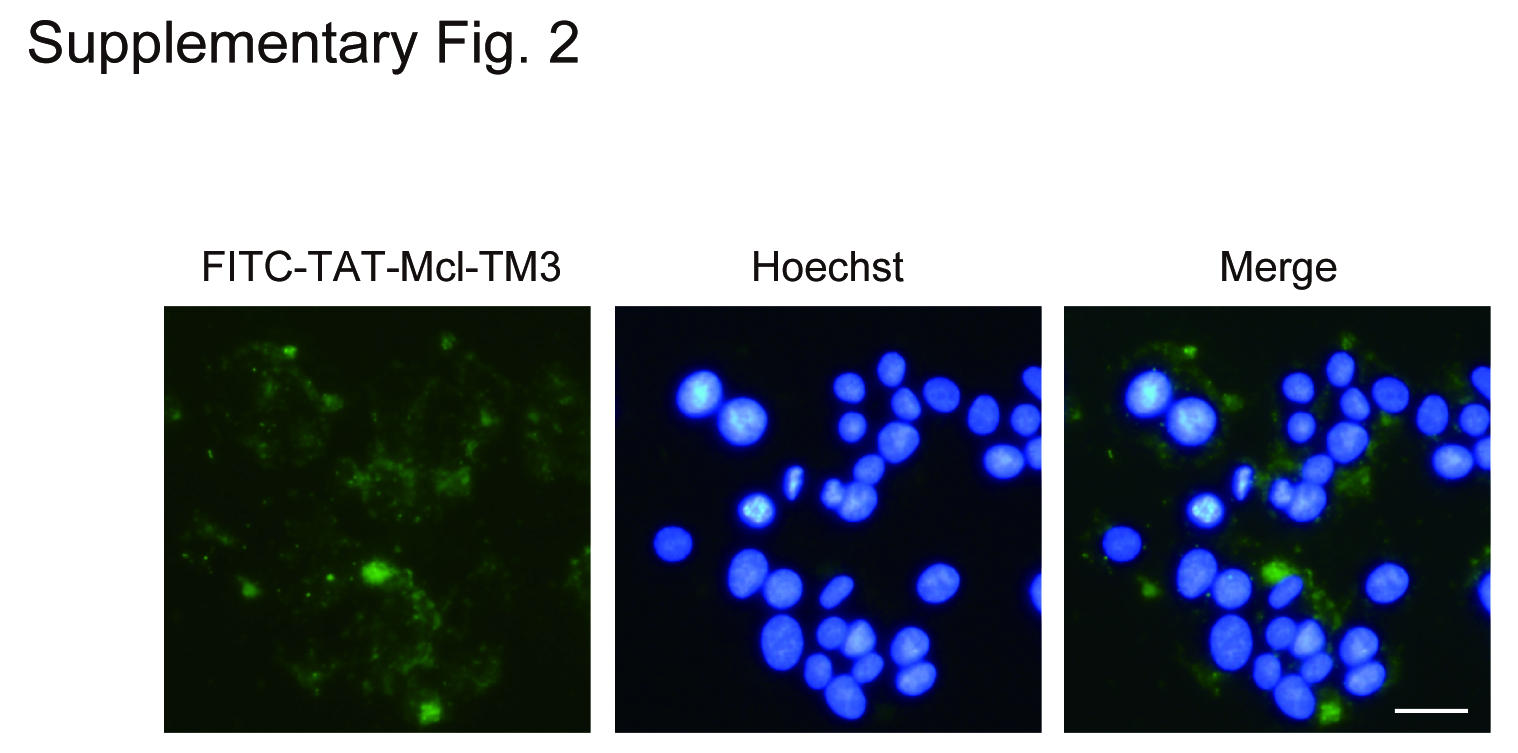

Supplement: Supplementary file 3 — Supplementary Figure 2 [file 41420_2021_475_MOESM3_ESM.tif]

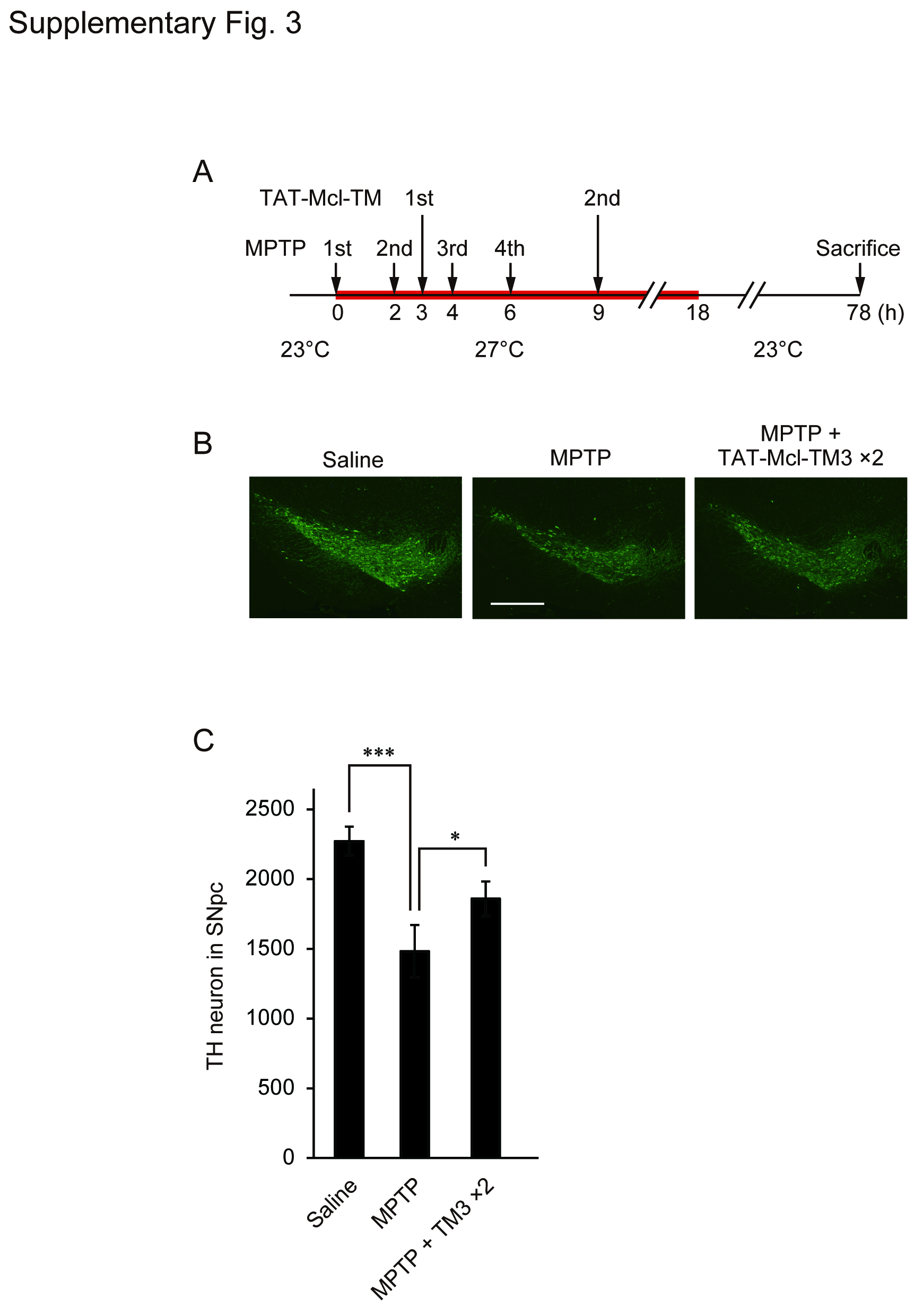

Supplement: Supplementary file 4 — Supplementary Figure 3 [file 41420_2021_475_MOESM4_ESM.tif]
